# Pneumocystis jirovecii Pneumonia Complicated by Pneumomediastinum: A Case Report

**DOI:** 10.7759/cureus.58189

**Published:** 2024-04-13

**Authors:** Pradeep Kumar Mada, Muhammad H Khan

**Affiliations:** 1 Infectious Diseases, Comanche County Memorial Hospital, Lawton, USA; 2 College of Osteopathic Medicine, Michigan State University, East Lansing, USA

**Keywords:** trimethoprim-sulfamethoxazole, influenza virus type a, pneumo mediastinum, pjp, hiv aids

## Abstract

Pneumomediastinum refers to the presence of air in the mediastinum (the space in the chest between the lungs). It can arise from various etiologies, including trauma, esophageal perforation, infections, medical procedures, or underlying lung diseases. *Pneumocystis jirovecii *pneumonia (PJP) is a common opportunistic infection seen in immunocompromised individuals, especially those with HIV/AIDS. Pneumomediastinum is a rare but serious complication of PJP that occurs in immunosuppressed patients, leading to significant morbidity and mortality. We present a rare case of pneumomediastinum caused by *P. jirovecii* pneumonia in an AIDS patient.

## Introduction

*Pneumocystis jirovecii* is an opportunistic fungus that can cause respiratory infections in immunosuppressed individuals, including a large number with AIDS [1]. In healthy, immunocompetent adults, *P. jirovecii* typically does not colonize the respiratory tract; however, there is increased susceptibility to colonization in patients with HIV, particularly in those with lower CD4 (helper T cells) counts [2-4]. Before the widespread use of antiretroviral therapy,* P. jirovecii* pneumonia (PJP) was one of the most common opportunistic infections in people with AIDS. It can cause severe respiratory symptoms, including cough, fever, difficulty breathing, and chest pain, and can be life-threatening if not treated promptly. With advances in antiretroviral therapy, the incidence of PJP has significantly decreased. However, it remains a concern, especially in people with poorly controlled HIV and who are on immunosuppressive medication such as high-dose steroids. Prophylactic antibiotics can be used for people with AIDS who have low CD4 cell counts to reduce the risk of developing PJP [1].

## Case presentation

A 25-year-old male with treatment-naive HIV presented to the emergency department with breathing difficulty. He had tested positive for HIV one year prior to admission and had declined treatment. He reported feeling unwell for three days with a productive cough, congestion, runny nose, body aches, progressively worsening shortness of breath, and chest tightness. He had a history of tobacco use, smoking one pack per day for five years. On arrival, the patient was found to be significantly hypoxic at 81% on room air. Arterial blood gas revealed a pH of 7.3, pCO2 of 58, pO2 of 54, and A-a gradient of 587, and the patient was found to be positive for influenza A (Table 1, Table 2).

**Table 1:** Complete blood count and comprehensive metabolic panel on admission GFR, glomerular filtration rate; HB, hemoglobin; MCH, mean corpuscular hemoglobin; MCHC, mean corpuscular hemoglobin concentration; MCV, mean corpuscular volume; MPV, mean platelet volume; RDW, red cell distribution width

Parameter	Result	Reference range	Unit
White blood cell count	10.4	4.40-11.00	10 × 3/mm^3^
Red blood cell count	4.91	4.50-5.90	10 × 6/mm^3^
Hb	14.3	13.2-16.5	g/dL
Hematocrit	42	39-49	%
MCV	85	80-94	fL
MCH	29	27-31	pg per cell
MCHC	34	33-37	g/dL
RDW	12.8	11.5-16.1	%
Platelets	153	130-440	10 × 3/mm^3^
MPV	9.2	7.2-11.1	fL
Neutrophil%	86	40-74	%
Lymphocyte%	6	19-48	%
Monocyte%	8	3.4-10.0	%
Sodium	133	135-145	mmol/L
Potassium	4.1	3.5-5.0	mmol/L
Chloride	94	96-110	mmol/L
Bicarbonate	26	21-31	mmol/L
Glucose	162	80-100	mg/dL
Blood urea nitrogen	8	6-21	mg/dL
Creatinine	0.71	0.6-1.4	mg/dL
Calcium	8.8	8.8-11.1	mg/dL
Total protein	7.6	5.9-8.4	g/dL
Albumin	4.4	3.2-5.2	g/dL
Total bilirubin	0.58	0.00-1.20	mg/dL
Alkaline phosphatase	78	41-133	u/L
Aspartate aminotransferase	21	7-39	u/L
Alanine aminotransferase	17	2-54	u/L
GFR	131	60-180	mL/min/1.73 m^2^

**Table 2:** Infectious workup PCR, polymerase chain reaction; RSV, respiratory syncytial virus

Parameter	Result
Influenza A by PCR	Positive
Influenza B by PCR	Negative
RSV infection by PCR	Negative
SARS-CoV-2 by PCR	Negative
Hepatitis C antibody	Negative
Hepatitis B surface antigen	Negative
Hepatitis B surface antibody	Negative
HIV-1/2 fourth-generation antibody/antigen	Positive

**Figure 1:**
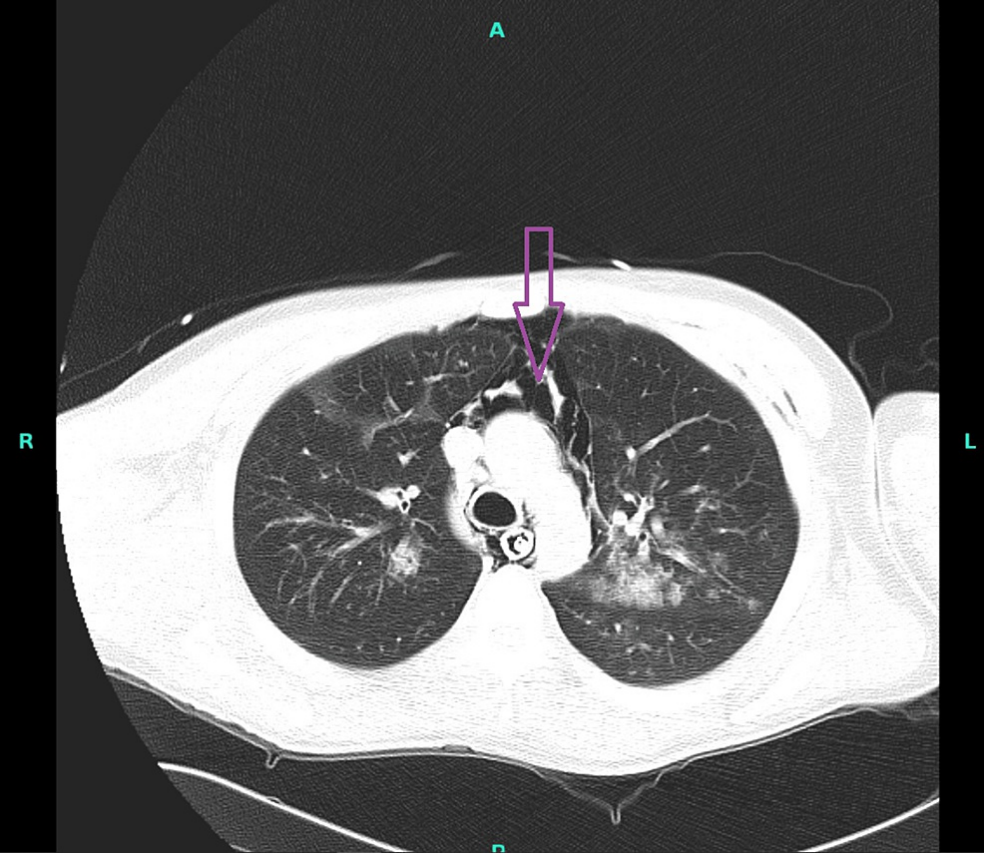
CT chest without intravenous contrast showing pneumomediastinum (purple arrow)

**Figure 2:**
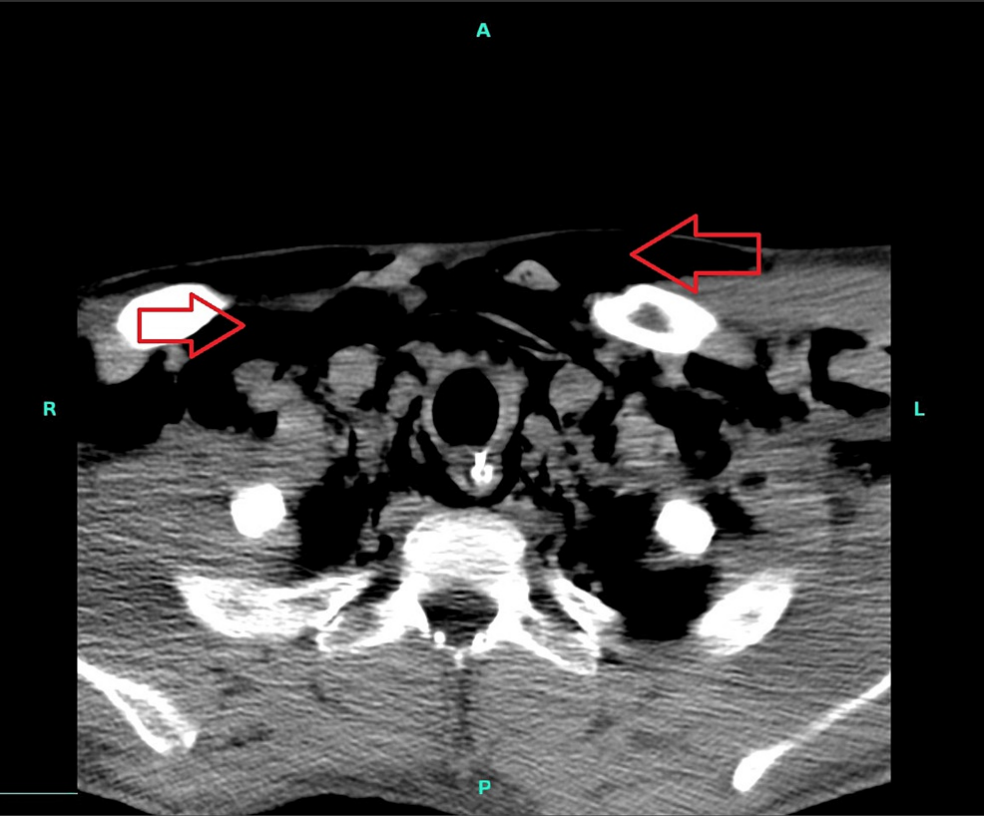
CT chest without intravenous contrast showing subcutaneous emphysema throughout the bilateral neck and upper chest wall (red arrows)

**Figure 3:**
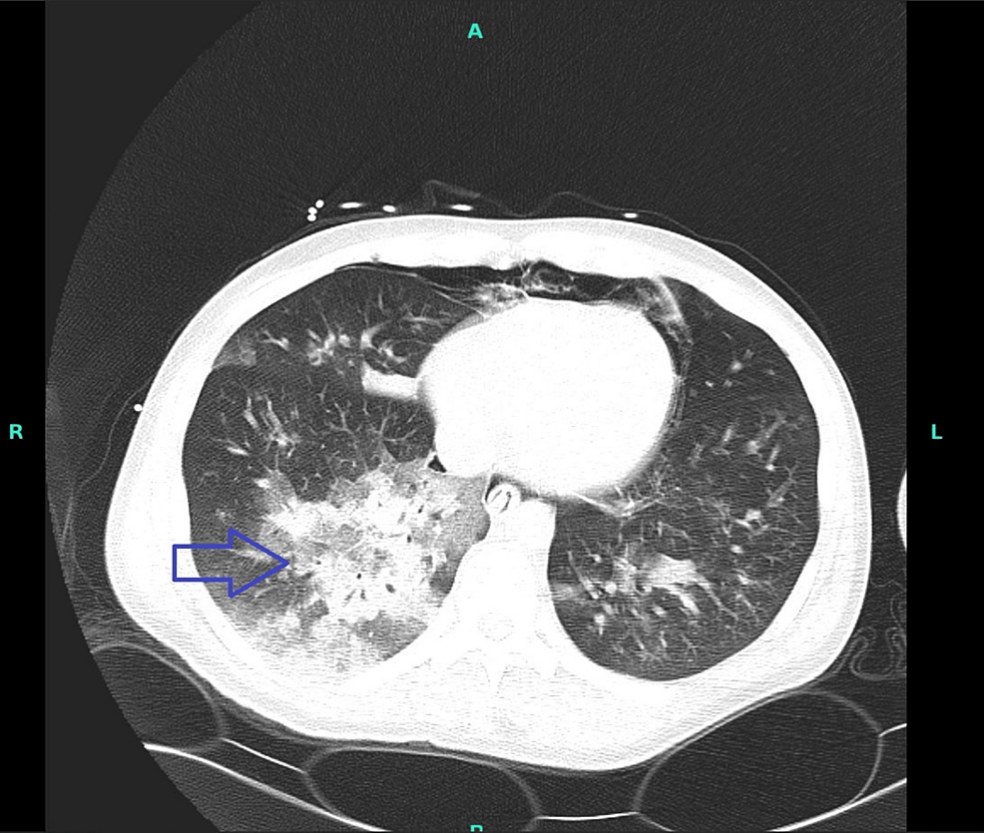
CT chest without intravenous contrast showing extensive bilateral centrilobular ground glass and consolidative airspace opacities throughout the lungs, bilaterally more pronounced in the right lower lobe (blue arrow)

The chest X-ray revealed right middle lobe pneumonia. The patient was intubated and placed on ventilator support. He was started on oseltamivir phosphate 75 mg twice daily for five days. On day 3 of admission, a physical exam revealed crepitus on the left side of the neck and chest. A CT scan of the thorax revealed extensive pneumomediastinum and subcutaneous emphysema throughout the bilateral neck and upper chest wall, as well as extensive bilateral centrilobular ground glass and consolidative airspace opacities throughout the lungs, with bilateral prominence, particularly in the right lower lobe (Figures 1-3).

Cardiothoracic and vascular surgery were consulted on the placement of a bilateral chest tube, and surgical intervention was not indicated. The HIV workup revealed a CD4 count of 83, consistent with AIDS. The HIV viral load was 2490. Since he was at risk of opportunistic infection with his CD4 count, he was empirically started on PJP treatment with IV trimethoprim-sulfamethoxazole (5 milligrams (mg)/kg three times daily trimethoprim + 25 mg/kg three times daily sulfamethoxazole) and a prednisone taper for a total of 21 days. The sputum PJP polymerase chain reaction was positive. He was also started on oral Biktarvy (Bictegravir 50 mg/emtricitabine 200 mg/tenofovir alafenamide 25 mg) (Table 3).

**Table 3:** HIV workup PCR, polymerase chain reaction

Parameter	Result	Reference range	Unit
% CD4+ (helper cells)	20	30-61	
Absolute CD4+ cells	83	490-1740	cells/uL
% CD8+ (suppressor T cells)	46	12-42	%
Absolute CD8+ cells	192	180-1170	cells/uL
Helper/suppressor ratio	0.43	0.86-5.00	
Absolute lymphocytes	413	850-3900	cells/uL
HIV-1 RNA quantitative PCR	2610	Not detected	copies/mL
HIV-1 RNA quantification PCR	3.42	Not detected	Log copies/mL

The patient showed significant improvement on treatment; he was subsequently extubated and discharged home on room air. An X-ray at the time of discharge showed that the lungs were adequately expanded without evidence of suspicious mass lesions or infiltrates, and the mediastinal structures and heart were within normal limits. At the six-month follow-up, his CD4 count was 503, and his HIV viral load was undetected.

## Discussion

The patient developed pneumomediastinum despite the use of low tidal volume and low airway pressure (plateau pressure: 22 ± 3 centimeters of water). Pneumomediastinum is a rare but noteworthy complication in patients with PJP [5]. Clinical manifestations of pneumomediastinum may include chest pain, dyspnea, coughing, and neck swelling [6]. Additionally, iatrogenic etiologies, such as intubation, have been implicated in instances of secondary pneumomediastinum [7]. It is postulated to result from alveolar rupture due to increased intrapulmonary pressure secondary to coughing, mechanical ventilation, or barotrauma in the setting of severe lung disease. In our case, the combination of severe PJP and coughing likely contributed to the development of pneumomediastinum.

Management of pneumomediastinum in PJP includes supportive care with oxygen supplementation, pain management, and treatment of the underlying infection. In severe cases, surgical intervention may be required. The prognosis depends on the severity of the underlying lung disease and the timely recognition and management of complications like pneumomediastinum. These include the action of proteolytic enzymes and the destruction of lung parenchyma by *P. jirovecii* result in cyst formation and interstitial fibrosis [8-11].

Pneumomediastinum is normally considered a benign entity, with management geared toward symptom relief [7]. However, when infectious processes underlie pneumomediastinum in populations with HIV, the prognosis is significantly worse [12,13]. Therefore, it is imperative to treat the underlying infection with antimicrobials and not simply aim for symptom relief, which would be done in noninfectious-related pneumomediastinum. Trimethoprim-sulfamethoxazole remains the most effective treatment for PJP, with the addition of corticosteroids lowering the cumulative risk of respiratory failure and death in those with AIDS. Furthermore, prophylaxis in patients with HIV/AIDS should be considered if the CD4+ count falls below 200 cells/μl [14].

## Conclusions

Pneumomediastinum can be a rare but serious complication of PJP, especially in immunocompromised patients. Clinicians should be vigilant in monitoring for this complication, especially in individuals with severe respiratory symptoms or worsening chest pain despite appropriate treatment for PJP. Early recognition and appropriate management are crucial in optimizing patient outcomes.
